# Full-Length Spatial Transcriptomics Reveals the Unexplored Isoform Diversity of the Myocardium Post-MI

**DOI:** 10.3389/fgene.2022.912572

**Published:** 2022-07-22

**Authors:** Etienne Boileau, Xue Li, Isabel S Naarmann-de Vries, Christian Becker, Ramona Casper, Janine Altmüller, Florian Leuschner, Christoph Dieterich

**Affiliations:** ^1^ Section of Bioinformatics and Systems Cardiology, Klaus Tschira Institute for Integrative Computational Cardiology, Heidelberg, Germany; ^2^ Department of Internal Medicine III (Cardiology, Angiology, and Pneumology), University Hospital Heidelberg, Heidelberg, Germany; ^3^ DZHK (German Centre for Cardiovascular Research) Partner Site Heidelberg/Mannheim, Heidelberg, Germany; ^4^ Cologne Center for Genomics (CCG), University of Cologne, Cologne, Germany; ^5^ Berlin Institute of Health at Charité-Universitätsmedizin Berlin, Max Delbrück Center for Molecular Medicine, Berlin, Germany

**Keywords:** spatial transcriptomics, single-cell RNA sequencing, oxford nanopore technology, myocardial infarction, visium spatial

## Abstract

We introduce Single-cell Nanopore Spatial Transcriptomics (scNaST), a software suite to facilitate the analysis of spatial gene expression from second- and third-generation sequencing, allowing to generate a full-length near-single-cell transcriptional landscape of the tissue microenvironment. Taking advantage of the Visium Spatial platform, we adapted a strategy recently developed to assign barcodes to long-read single-cell sequencing data for spatial capture technology. Here, we demonstrate our workflow using four short axis sections of the mouse heart following myocardial infarction. We constructed a *de novo* transcriptome using long-read data, and successfully assigned 19,794 transcript isoforms in total, including clinically-relevant, but yet uncharacterized modes of transcription, such as intron retention or antisense overlapping transcription. We showed a higher transcriptome complexity in the healthy regions, and identified intron retention as a mode of transcription associated with the infarct area. Our data revealed a clear regional isoform switching among differentially used transcripts for genes involved in cardiac muscle contraction and tissue morphogenesis. Molecular signatures involved in cardiac remodeling integrated with morphological context may support the development of new therapeutics towards the treatment of heart failure and the reduction of cardiac complications.

## Introduction

Cell type heterogeneity has recently emerged as a major aspect in redrawing the cellular picture of the mammalian heart (Litviňuková et al., 2020; Tucker et al., 2020; Wang et al., 2020). Single-cell RNA-seq (scRNA-seq) technology has enabled to explore crosstalk of different cardiac cell populations to identify response signatures involved in remodeling after myocardial infarction (MI) and ischemic injury (Cui et al., 2020; Forte et al., 2020; Ruiz-Villalba et al., 2020; Vafadarnejad et al., 2020; Gladka et al., 2021; Heinrichs et al., 2021; Molenaar et al., 2021; Tombor et al., 2021). These and other data provide a valuable compendium of information to better understand transcriptional changes occuring in cardiomyocyte and non-cardiomyocyte sub-populations in healthy, injured, and regenerating hearts. However, in all these studies, the original tissue architecture is destroyed and, in general, the morphological context is lost, including the relationship of cells to infarct, border and remote zones (van Duijvenboden et al., 2019).

Recent development in spatial transcriptomics addresses this challenge, but few studies only have provided spatially resolved insights into the cardiac transcriptome. Techniques such as microdissection (Wu et al., 2016; Burkhard and Bakkers, 2018) or, in particular, spatially barcoded arrays and *in situ* capturing, have enabled to retain the spatial information while profiling the whole transcriptome at near-single-cell resolution, allowing to shed light on localized tissue neighbourhoods (Asp et al., 2017, 2019), but a detailed characterization of cellular zones of injury, repair and/or remodeling is lacking. MI is a complex spatio-temporal heterogeneous disease involving the whole heart, and unbiased spatial transcriptomics holds a promise to add tissue context to molecular profiling in the search of novel therapeutics.

One of the most established spatial transcriptomics methods is now widely available as the Visium platform by 10x Genomics. After the tissue section is fixed on the spatial slide, stained, and imaged, it is permeabilized to release RNA to bind to capture probes for on-slide cDNA synthesis. Library preparation is performed off-slide, and spatial barcodes and tissue image are used to overlay transcriptomics data with tissue context. Although several new such methods have recently been published (Rodriques et al., 2019; Liu et al., 2020), they are for the most part relying on short-read library preparation. Thus they fail to generate sufficient overlaps to reconstruct transcriptomes *de novo*.

Adequate gene and transcript models are instrumental towards relevant proof of concepts and investigational new drug development in translational cardiac research (Müller et al., 2021). Third-generation or long-read sequencing allow to reconstruct truthful assemblies with fewer gaps and to characterize complete transcript isoforms and chimeric transcripts. This has recently been taken to the single-cell level using either Pacific Biosciences (PacBio) or Oxford Nanopore technology (Gupta et al., 2018; Lebrigand et al., 2020; Joglekar et al., 2021; Volden and Vollmers, 2021; Wang et al., 2021).

Here, taking advantage of the conceptual similarity between spatial and cell barcodes, we introduce Single-cell Nanopore Spatial Transcriptomics (scNaST), a set of tools to facilitate whole transcriptome spatial profiling of full-length transcripts, based on a previously published Bayesian approach for cell barcode assignment (Wang et al., 2021). Our method relies on the commercially available Visium platform by 10x Genomics and Nanopore long-read sequencing, although it can in principle be extended to other technologies, as long as a hybrid sequencing approach is used for spatial barcode assignment.

In this short report, we demonstrate how scNaST can be used to characterize the spatial transcriptional landscape of the heart post-MI. Using our workflow, we were able to assign a total of 7,616 spatial spots across four short axis sections of the heart, corresponding to distinct 19,794 transcript isoforms in total, encoded by 12,474 genes. We showed a higher transcriptome complexity in the healthy regions, and identified intron retention as a mode of transcription associated with the injured areas. Our results showed a clear regional isoform switching among differentially used transcripts for genes involved in cardiac muscle contraction and tissue morphogenesis, many of them clinically relevant, opening new opportunities in translational cardiac research.

## Materials and Methods

### Experimental Model of Myocardial Infarction

A C57BL/6 mouse (female, 8 weeks old, Janvier Labs) was exposed to 5% isoflurane for anesthesia. An intubation cannula was inserted orally into the trachea. The mouse was fixed on a heating plate at 37°C and maintained under anesthesia with 2% isoflurane. An incision was made from the left sternum to the midclavicular line. Skins and muscle layers were stretched with forceps and sutures. Another incision was made between the third and fourth intercostal space. The heart was exposed and subjected to permanent myocardial infarction with ligation of the left anterior descending (LAD) coronary artery. When the ribs and skins were fully closed, the isoflurane supply was cut off. Oxygen was then supplied until normal breathing was resumed.

### Heart Extraction and Cryosection

Three days after permanent LAD ligation, the mouse was sacrificed for organ harvest. After washing with cold PBS several times to remove the blood, the heart was transferred into a bath of isopentane (Millipore Sigma) frozen by liquid nitrogen. The freshly obtained heart was kept fully submerged in isopentane for 5 min until fully frozen. Pre-cooled Cryomold on the dry ice was filled with chilled TissueTek OCT compound without introducing bubbles. The frozen tissue was then transferred into the OCT with pre-cooled forceps and placed on the dry ice until the OCT was completely frozen. OCT-embedded tissue blocks were removed from the Cryomold and mounted on the specimen stage. 10*μ*m sections were cut in a cryostat at -10°C and placed within a Capture Area on the pre-equilibrated Visium Spatial Slides (10x Genomics). The slides were later sealed in individual 50 ml Falcon at -80°C ready for further processing.

### 10X Genomics Visium Experiments

Four short axis sections of the heart were processed according to the manufacturer’s protocol. Libraries were prepared individually, one per heart section. The Visium Spatial Tissue Optimization Slide & Reagent kit (10x Genomics) was used to optimize permeabilization conditions. Tissue permeabilization was performed for 24 min. Spatially barcoded full-length cDNA was generated using the Visium Spatial Gene Expression Slide & Reagent kit (10x Genomics). A fraction of each cDNA library was used for Nanopore sequencing. cDNA amplification was then conducted for 20 cycles of PCR (identified by qPCR), and 400 *μ*l were used in the 10xGenomics Visium library preparation (100 *μ*l per section). The libraries were sequenced on a NovaSeq6000 (Illumina), with 29 bases from read 1 and 90 bases from read 2, at a depth of 160M reads per section (640M reads in total). The raw sequencing data was processed with the 10x Genomics Space Ranger v1.1.2 and mapped to the mm10 genome assembly (mm10-2020-A).

### Oxford Nanopore Sequencing Libraries

Libraries for Nanopore sequencing were prepared according to the manufacturer’s protocol for direct cDNA Sequencing (SQK-DCS109 Oxford Nanopore Technologies) with the following minor modifications: The protocol was started with 200 ng cDNA with the End-prep step. Final library elution was performed in 15 *μ*l to have material left for TapeStation analysis and Qubit BR measurement. Four GridIon flow cells (FLO-MIN106) were loaded with 12 *μ*L libraries (74–115 ng) by using a Flow Cell Priming kit EXP-FLP002. Base calling was done with Guppy v5.0.12. The High accuracy (HAC) model was selected for base calling (Q-Score cut-off > 9).

### Spatial Barcode Assignment

To account for source-specific quality differences, each heart section (Illumina libraries) was processed separately using Scanpy v1.7.2 (Wolf et al., 2018), keeping only spatial barcodes with approximately 150 < counts < 18,000, 250 < genes < 5,000, detected in at least two spots, and with less than 40% mitochondrial counts. The resulting datasets were concatenated, normalized, and the union of highly variable genes (per section) were kept for final analysis. Batch balanced KNN (BBKNN) (Polański et al., 2020) with ridge regression (Park et al., 2020) was used for integration and batch correction, starting from a coarse clustering obtained from a BBKNN-corrected graph.

For the Nanopore libraries, samples were demultiplexed and processed with ScNapBar v1.1.0 (https://github.com/dieterich-lab/single-cell-nanopore) using a naive Bayes model to assign spatial barcodes (Wang et al., 2021). Briefly, for each heart section, the Space Ranger results (Illumina libraries) were used to parametrize a model of barcode alignment features to discriminate correct versus false barcode assignment in the Nanopore data. FASTQ files were mapped using minimap2 v2.21 (Li, 2018). For transcript isoform quantification, a *de novo* transcriptome annotation was generated. Alignment files were processed by StringTie v2.1.5 in long read mode with the reference annotation to guide the assembly process (Kovaka et al., 2019). The annotations were merge into a non-redundant set of transcripts and compared to the reference using GffCompare v0.12.2 (Pertea and Pertea, 2020), after removing single-exon transcripts. To generate feature-spatial barcode matrices, alignment files were split into multiple files, one per spatial barcode, based on the barcode assignments, converted to FASTQ, and aligned to the *de novo* transcriptome with minimap2. Abundances were quantified with Salmon v1.5.2 in alignment-based mode using a long read error model (Patro et al., 2017). Each section was processed separately using Scanpy v1.7.2 and integrated with BBKNN, as described above for the Illumina data. Spatial barcodes were filtered for counts (approximately 50 < counts < 4,000), transcripts (approximately 50 < transcripts < 2000, detected in at least two spots), and ribosomal genes ( < 40%). Neighbors enrichment and cluster co-occurence analyses were performed using Squidpy (Palla et al., 2021).

### Identification of Anatomical Regions

For the Illumina libraries, the neighborhood graph was computed using BBKNN. Spots were clustered with a low resolution (0.3) to identify anatomical regions such as infarct, border and remote zones. Marker genes were identifed using a Wilcoxon rank sum test with Benjamini–Hochberg correction, by comparing the expression of each gene in a given cluster against the rest of the spots. The final clusters were manually annotated.

Labels were then assigned to Nanopore spatial barcodes based on the set of matching Illumina barcodes. However, not all assigned barcodes were labeled due to quality control filtering criteria that were different between Illumina and Nanopore datasets. To assign labels to the remaining Nanopore barcodes, seed labelling was performed with scANVI using the set of assigned labels as groundtruth (Xu et al., 2021). The top expressed transcripts were then identified as described above for the Illumina data.

### Spatial Spot Deconvolution

Spatial spots were deconvoluted using stereoscope (scvi-tools v0.15.0) (Andersson et al., 2020). Heart data (Smart-Seq2 and 10x Genomics) from the Tabula Muris (Schaum et al., 2018) were used as reference dataset and highly variable genes were identified. For the Smart-Seq2 data, gene length normalization was applied. The model was trained on the single cell reference dataset on the intersection of genes found in the spatial (Illumina) data, and proportions were inferred for each Visium spot for each cell type in the reference dataset. Labels were then assigned to Nanopore spots are described above.

### Spatial Gene Expression (Illumina)

For each heart section, genes with spatial expression patterns were obtained with SPARK (FDR < 0.05) (Sun et al., 2020). Overrepresented biological processes in each region were identified using spatially variable markers that were previously identified (logFC < 0.2, *p*-value < 0.05), using Enrichr with the Python package GSEApy.

### Differential Transcript Usage (Nanopore)

To identify changes in relative usage of transcripts/isoforms within genes, differential transcript usage (DTU) tests were performed using satuRn (Gilis et al., 2021). Only multi-exon transcripts and genes with more than one isoform were kept for the analyses. The transcript count matrix was further filtered to keep transcripts expressed in a worthwhile number of spots, determined by the design (but greater than at least 10% of the smallest group size), with a CPM count above a threshold of 1(median library size)^−1^. In addition, transcripts were kept only if they had a minimum count of one across all spots. A quasi-binomial generalized linear model was fit using a design comparing each anatomical region with another, or each anatomical region with the rest of all regions. A two-stage testing procedure was performed using stageR (Van den Berge and Clement, 2021), with an OFDR of 0.05. Results were reported using a student’s t-test statistic, computed with estimated log-odds ratios. To identify isoform-switching genes, significant genes were identifed for each contrast with at least two transcripts showing a switching expression pattern between regions of interest.

### Transcript Classes

Transcript classes were assigned in scNaST using Gffcompare (Pertea and Pertea, 2020). For the identification of isoforms and transcript classes between regions, markers previously identifed were used to select transcripts in each region with a logFC > 0.1 (*p*-value < 0.05). Enrichment of transcripts of a certain class (*e.g.* intron retention) was calculated using a Fisher exact test, using only non-equality classes. Motif enrichment was performed using Simple Enrichment Analysis (SEA) from the MEME suite, using retained intron sequences vs. annotated (reference transcript) sequences (Bailey et al., 2015).

### RT-PCR Analysis of Isoform Expression

Total RNA was isolated from the heart of Sham (n = 3) or infarcted mice (n = 3, 3 days post-MI) according to standard methods. In the latter case, RNA from the infarct (including the border zone) and the remote zone was isolated separately. For each sample, 1 *μ*g total RNA was reverse transcribed usind Maxima First Strand cDNA synthesis kit (Thermo Fisher Scientific, Waltham, MA, United States) and Random Primers according to the manufacturer’s protocol. Prior to cDNA synthesis, RNA was treated with DNase included in the kit. cDNA was diluted 1:1 with nuclease-free water and stored at -20°C. The specific amplification of Pdlim5, Actc1 and Tpm1 isoforms as indicated in Supplementary Figure S7 was carried out with primers (Supplementary Table S4) and GoTaq G2 Flexi DNA Polymerase (Promega, Madison, WI, United States). 25*μ*l reactions contained 1 *μ*l diluted cDNA, 1x Taq buffer green, 0.4 mM dNTPs, 1.5mM MgCl_2_, 0.1 *μ*l GoTaq and 0.4 *μ*M forward and reverse primer, respectively. PCR reactions were carried out as follows: Initial denaturation for 3 min at 94°C, followed by 20–35 cycles of 30 s denaturation at 94°C, 30 s annealing at 57°C, 90 s elongation at 72°C. After a final elongation (2 min, 72°C) samples were cooled until analysis. 5*μ*l PCR products were analyzed on a 2% agarose gel in TBE that was stained with GelRed (Biotium, Freemont, CA, United States) and images were acquired with a ChemiDoc instrument (BioRad, Hercules, CA, United States). The size of the PCR products was estimated based on the migration behaviour of GeneRuler 50 bp ladder (Thermo Fisher Scientific, Waltham, MA, United States).

## Results

### scNaST Enables the Demarcation of Spatially Distinct Regions of the Myocardium Post-MI

**FIGURE 1:**
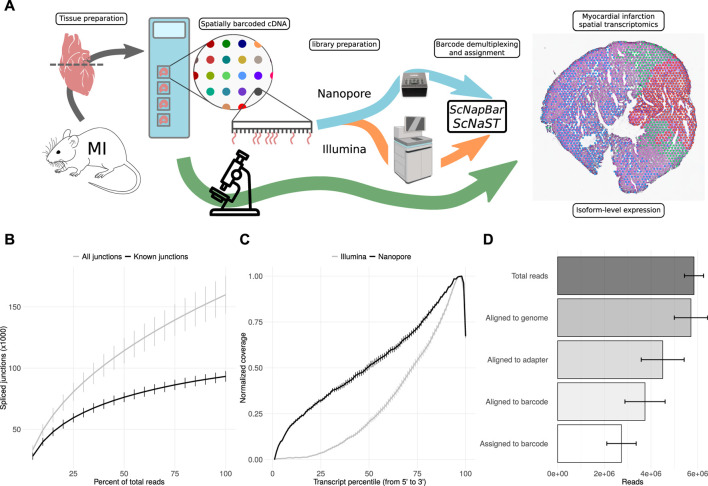
scNaST methodology. **(A)** Schematic of the scNaST workflow using a hybrid sequencing approach on Nanopore and Illumina platforms to assign spatial barcodes to long-read sequencing. **(B)** Nanopore sequencing saturation showing the number of splice sites detected at various levels of subsampling. A curve that reaches a plateau before getting to 100% data suggest that all known junctions in the library have been detected. The curve shows the mean ± SE of four samples. **(C)** Normalized transcript coverage for Nanopore and Illumina. The curves show the mean ± SE of four samples. **(D)** Reads assigned by scNapBar at each step of the workflow. The bars show the mean ± SE of four samples.

Fresh-frozen tissue samples were stained, imaged and fixed on Visium Spatial Gene Expression Slides (10X Genomics) for permeabilization and *in situ* RNA capture. Full-length cDNA libraries were split for the preparation of 3’ Illumina short-read and direct long-read Nanopore sequencing libraries. Short-read data were used for the assignment of spatial barcodes to Nanopore reads using the scNapBar workflow, and subsequently used to define anatomical regions within the tissue organization (Figure 1A). Long-read data were used for transcriptome assembly and transcript abundance quantification, and layered onto the stained images to reveal the spatial organization of isoform expression. The Nanopore data comprises of four heart slices (or samples) with a total of 25,5 million reads, reaching a relatively high sequencing saturation (Figure 1B), and providing a significant gain in coverage along full-length transcripts (Figure 1C). After spatial barcode assignment, libraries had a median of 2.8 million reads per sample, with over 70% assigned reads (Figure 1D and Supplementary Figure S1A). Despite variations across samples, we observed a good per spot correlation between Illumina and Nanopore libraries (Supplementary Figure S1B,C). Per spatial spot (55 *μ*m), after quality filtering, we observed a median read count varying between 593 and 2021 corresponding to a median of 311 (respectively 1,000) distincts isoforms (Supplementary Figure S2, see also Supplementary Figure S3 Supplementary Figure S4).

**FIGURE 2:**
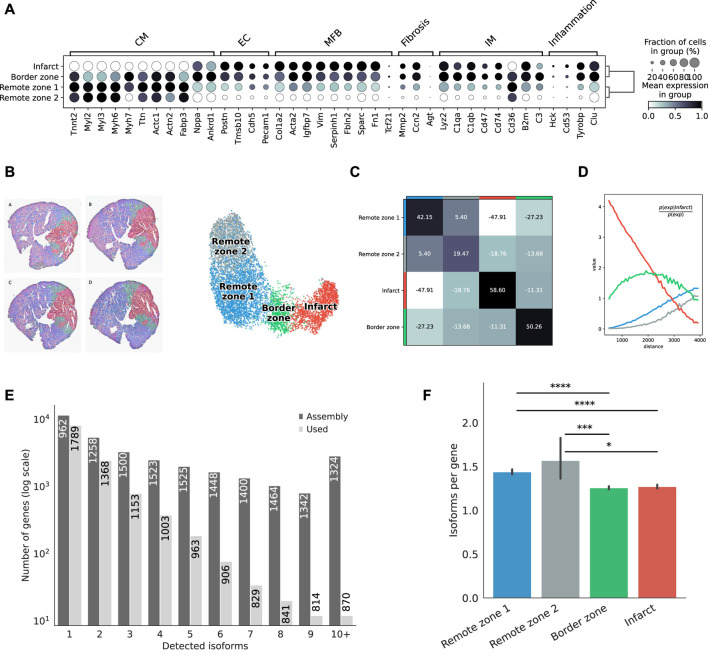
Defining morphological regions after MI. **(A)** Dot plot showing the expresson of selected markers associated with the expresson of CM = cardiomyocytes, EC = endothelial cells, MFB = myofibroblasts, IM = immune cells, or with fibrosis and inflammation, based on the short-read Illumina data. **(B)** Annotation of mouse heart regions after MI via short-read clustering, transfered to the Nanopore data. Scatter plot in spatial coordinates of the anatomical regions (left) and UMAP representation of the Nanopore data using the region annotation from short-read clustering (right). Colors in the spatial scatter plot are matching those of the UMAP. **(C)** Neighbors enrichment analysis in one heart axis section. The heatmap shows the enrichment score on spatial proximity between the different anatomical regions. Spots belonging to two different regions that are close together will have a high score, and vice-versa. **(D)** Cluster co-occurrence in spatial dimensions in one heart axis section. Line plot showing the conditional probability of observing a given region conditioned on the presence of the infarct region, computed across increasing radii size around each spots. Distance units are given in pixels of the Visium source image. **(E)** Barplot showing the frequency distribution of the number of isoforms per gene, either stemming from the assembly, or found in the final data after quality control filtering. The median length of transcripts is indicated in each bar for each category. **(F)** Average number of isoform per gene detected among markers of each morphological region. Significance was measured using a Mann-Whitney U-test (*** = < 0.001, **** = < 0.0001)

Clustering based on short-read gene expression defined four broad morphological regions: two remote zones that stemmed from differences in sequencing depth between heart slices, a border zone, and the infarct. Remote zones and, to a lesser extent the border zone, were largely associated with cardiomyocyte markers, while the border and infarct zones were characterized by a higher expression of endothelial, myofibroblast, and immune marker genes, as well as with markers of fibrosis and inflammation (Figure 2A). The region classification was then transfered to the Nanopore data (Figure 2B), and markers of each region were identified (Supplementary Figure S5). We also investigated, using the Illumina libraries, whether our spatial transcriptomics data reflected known biological processes of myocardial infarction. We identified spatially variable genes across samples and characterized each region using biological processes (Supplementary Figure S6, and supporting information Supplementary Table S1). Remote zones were generally associated with cardiac muscle contraction linked to an overrepresentation of healthy cardiomyocytes, and included genes such as Strit1 (SERCA regulator DWORF), cardiac troponin I (Tnni3), myosin-binding protein C (Mybpc3), or ventricular myosin light chain-2 (Myl2). The border zone was characterized by genes such as Nppb or Ankrd1, both of which were reported to be upregulated in the border zone after MI (Hama et al., 1995; Mikhailov and Torrado, 2008), and other genes associated with the complement system and myogenesis. Overrepresented processes were however similar to those found in the infarct area. Top markers of the infarct area included collagens and genes associated with TGF-*β* and p53 signalling, with hypoxia, coagulation, epithelial to mesenchymal transition, and the extracellular matrix. Overrepresented processes included neutrophil degranulation, and gene expression associated with the innate immune system. Remote, border, and infarct zones had a clear distinct spatial organization: the two remote zones co-occured at short distances with one another, but did not show any neighborhood enrichment with the border and infarct zones (Figures 2C,D). We observed a slight co-enrichment of the infarct and the border zone at medium distances (Figure 2D), revealing how spatial resolution may, in general, affect morphological classification.

Following this strategy, we successfully assigned 7,616 spatial spots, corresponding to distinct 19,794 transcript isoforms in total, encoded by 12,474 genes. Among all genes, we observed 8,131 (67,3%) that expressed a single isoform and 3,953 (32.7%) that expressed two or more isoforms (Figure 2E). Although predicted by our assembly, genes with many isoforms were expressed at a lower threshold and were not included in our final analyses. We observed variations in the number of isoforms per gene across morphological regions of each heart slice, with significant differences between either of the remote zones and the border and the infarct areas, suggesting the existence of a higher transcriptome diversity in the healthy regions (Figure 2F).

### scNaST Reveals the Spatial Isoform Diversity of the Myocardium Post-MI

**FIGURE 3:**
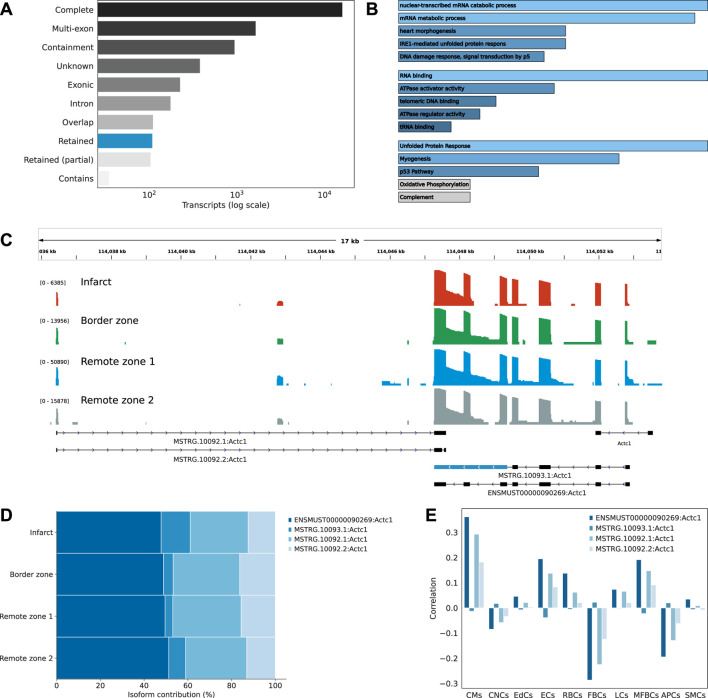
Characterizing the isoform diversity after MI. **(A)** Barplot showing how full-length transcripts obtained with scNaST compare to the existing mouse annotation. Labels Complete (=), Multi-exon (j), Containment (k), Unknown (u), Exonic (x), Intron(i), Overlap (o), Retained (m, n), and Contains (y) are explained in https://ccb.jhu.edu/software/stringtie/gffcompare.shtml. **(B)** Over-representation analysis of genes harboring novel transcripts with intron retention (IR). From top to bottom, biological processes, molecular function, and hallmark gene sets from the Molecular Signatures Database (MSigDB). **(C)** Exonic structure of the different Actc1 isoforms, including novel isoforms identified by scNaST with IR and exonic antisense overlap. Coverage (log scale) is shown for each region with a different scale. **(D)** Actc1 isoform contributions to total Actc1 expression in the different heart regions. **(E)** Per spot correlation observed between spatial deconvolution of cell types and Actc1 isoforms.

Transcript isoforms were largely associated with exact matches to the reference annotation, multi-exon transcripts with at least one junction match to the reference (*e.g.* exon skipping and exon extension), transcripts longer than the reference (containment of reference), completely novel transcripts (intergenic), transcripts with exonic overlap, intronic transcripts, or retained introns (Figure 3A). Among these classes, we were particularly interested in novel transcripts associated with retained introns. Intron retention (IR) occurs when an intron is transcribed into pre-mRNA and remains in the final mRNA. Only recently has IR become of interest due to its associations with complex diseases (Zhang et al., 2020; Boeckel et al., 2021). Interestingly, among differentially expressed transcript markers, we found across the infarct and border zones 65 distinct IR transcripts, compared to 1,612 in the remote zones, corresponding to an odds ratio of 1.73 (*p*-value 0.008). When considering the infarct area only, the odds were even greater (odds ratio 2, *p*-value 0.002). Introns that were retained across the infarct and border zone were enriched in motifs associated with poly(C)-binding protein one and 3 (Pcbp1/3), KH RNA-binding domains (Khdrbs1), and Rbm38, a homolog of Rbm24, a pivotal cardiac splice factor (Weeland et al., 2015) (supporting information Supplementary Table S2). The genes harboring these transcripts were enriched in RNA metabolic/catabolic processes, RNA binding, and the unfolded protein response (Figure 3B). Among these, we found cardiac muscle alpha actin (Actc1), the major protein of the thin filament responsible for cardiac contraction (Figure 3C). Our data revealed a relatively high expresion of the IR transcript isoform across all regions, but a comparatively greater contribution to the infarct area (Figure 3D). These observations were confirmed by RT-PCR analysis (Supplementary Figure S7A). At the Actc1 locus, we also identifed two novel antisense overlapping transcripts with a good coverage across all regions (Figure 3C). Using the Tabula Muris (Schaum et al., 2018) as a reference dataset to perform deconvolution, we identified cardiomyocytes, and to a lesser extent, endothelial and myofibroblasts as the predominant origin of these novel antisense isoforms, with a correlation pattern that matched that of the largest Actc1 annotated isoform (Figure 3E). The expression of these novel transcripts was also confirmed by RT-PCR analysis (Supplementary Figure S7A).

### scNaST Allows to Study Regional Isoform Switching With Spatial Context

**FIGURE 4:**
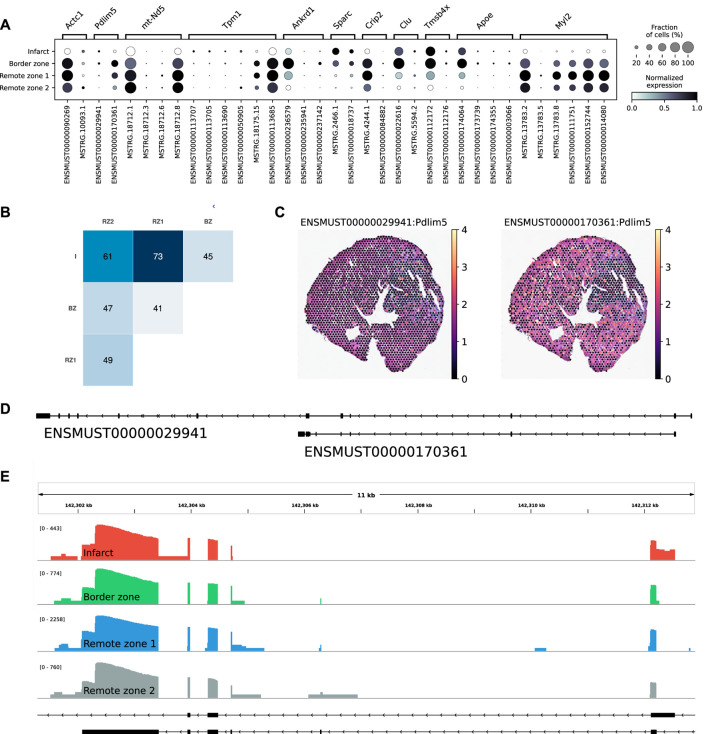
Regional isoform switching after MI. **(A)** Dot plot showing the top five most significant isoform-switching genes per contrast across all comparisons between the remote, border, and infarct areas, using a stage-wise testing procedure at an overall false discovery rate (OFDR) of 0.05. The top genes were restricted to the spatially variable genes. **(B)** Heatmap representing the number of isoform-switching genes identified between any two regions. I = infarct, BZ = border zone, RZ = remote zone. **(C)** Spatial scatter plot showing Pdlim5 isoform expression in one heart axis section. **(D)** Pdlim5 isoforms track. **(E)** Zoom of D to show coverage of both isoforms across regions.

Among the multi-isoform genes, we looked for those showing a splicing pattern variation across regions. We identified 109 significant regional isoform switching genes across all comparisons between the remote, border, and infarct areas (OFDR 0.05 at both gene and transcript level, supporting information Supplementary Table S3). Our results showed a clear regional isoform switching among differentially used transcripts for genes involved in cardiac muscle contraction and tissue morphogenesis, many of them clinically relevant, such as Tpm1 (England et al., 2017), Ankrd1 (Mikhailov and Torrado, 2008), Sparc (McCurdy et al., 2011), or Clu (Turkieh et al., 2019) (Figure 4A). The largest number of isoform-switching genes were found between the infarct and the remote zones (Figure 4B). Among them, we found PDZ and LIM domain protein 5 (Pdlim5), a gene encoding for a protein that localizes to the Z-disk by binding to *α*-actinin, and which has been implicated in dilated cardiomyopathy (Verdonschot et al., 2020) and in heart failure with preserved ejection fraction (Soetkamp et al., 2021). Pdlim5 has several splice variants, clustered into long and short isoforms, which are dynamically regulated during heart development (Yamazaki et al., 2010). The protein contains a PDZ domain at its N-terminus and, at its C-terminus, three LIM domains that are absent from the short isoforms. Our data showed a significant short-to-long isoform switch of two annotated variants between the border or the remote zones and the infarct (Figures 4C–E). The shorter isoform was mostly found in the border and remote zones, while the longer isoform was significantly, albeit at lower levels, expressed in the infarct area (Figures 4C,E and Supplementary Figure S7B. We also validated the expression of another short-to-long isoform switch in the Tpm1 gene, where the short unannotated variant is a novel isoform identified by scNaST (Supplementary Figure S7C).

## Discussion

scNaST expands the spatial transcriptomics toolbox to third-generation long-read sequencing. scNaST is based on commercially available platforms, and can be used to investigate the isoform landscape of complex tissue. We presented here a transcriptome-wide approach to explore annotated and *de novo* isoform diversity with morphological context in the mouse heart after myocardial infarction (MI). Our approach opens-up new opportunities to understand the spatial and molecular organization of the heart following injury. Although previous studies showed transcriptional differences between proximal and distal areas to the infarct (van Duijvenboden et al., 2019), observations were limited to conventional short-read sequencing approaches or were limited in spatial resolution.

Our results not only reflected known biological processes of MI, but also revealed the spatial organization of gene expresion and transcriptome diversity consistent with the underlying biological condition. Intron retention (IR) is a typical exemplar of regulated splicing which has been, until recently, largely overlooked in mammalian organisms (Jacob and Smith, 2017). In heart failure patients, increased IR of essential cardiac constituents such as Titin, is hypothesized to be involved in the cellular stress response (Boeckel et al., 2021), which would be consistent with our observations. There is also growing evidence that antisense-mediated gene regulation is involved in different pathophysiological contexts, including heart disease (Luther, 2005; Zinad et al., 2017; Celik et al., 2019). Recently, a long non-coding RNA transcribed antisense near the Actc1 gene was described for its role in cardiomyocyte proliferation and cardiac repair (Ponnusamy et al., 2019).

Finally, we identified regional isoform switches that could play a role in the myocardium post-MI. Among these, we found Tropomyosin 1 (Tpm1), a key contractile protein that undergoes extensive splicing in both human and mouse, and which is known to be involved in heart development and in the formation of congenital heart defects (England et al., 2017), and Pdlim5. The role of Tpm1 as an inhibitor of the actin-myosin interaction may suggest that isoform switching is associated with compensatory contractile mechanisms in MI, although this hypothesis remains entirely speculative. In a recent study, Pdlim5-short variants were shown to cause cytokinesis defects in post-natal cardiomyocytes (Gan et al., 2022). While long transcript expression gradually decreased after birth, the expression of short variants, initially low, increased from post-natal day 1, and the temporal switching was associated with the onset of cardiomyocyte binucleation. Although, in the context of MI, the biological significance of this switch remains elusive, it is tempting to explain the observed short-to-long ratio between the remote and the infarct zones with fetal gene re-expression, typically associated with metabolic remodeling under pathophysiologic conditions (Taegtmeyer et al., 2010). While further work is required to corroborate these observations, we envision that our effort will serve as a reference for future developments and studies integrating long-read sequencing with spatial gene expression data.

## Data Availability

The datasets generated for this study have been deposited in NCBI’s Sequence Read Archive through the BioProject accession number PRJNA843953. Additional information and scripts to generate the data and figures are available at https://github.com/dieterich-lab/ScNaST. Additional supporting data is available at https://doi.org/10.5281/zenodo.6546361. ScNapBar is publicly available at: https://github.com/dieterich-lab/single-cell-nanopore. ScNaST is publicly available at: https://github.com/dieterich-lab/ScNaST.
