# Supplementary material for: Full-Length Spatial Transcriptomics Reveals the Unexplored Isoform Diversity of the Myocardium Post-MI
Source: Front Genet. 2022 Jul 22;13:912572. doi: 10.3389/fgene.2022.912572 (PMC9354982; doi:10.3389/fgene.2022.912572)
Supplement: Supplementary file 3 [file DataSheet1.pdf]

frontiers

Supplementary Material

1 SUPPLEMENTARY TABLES AND FIGURES

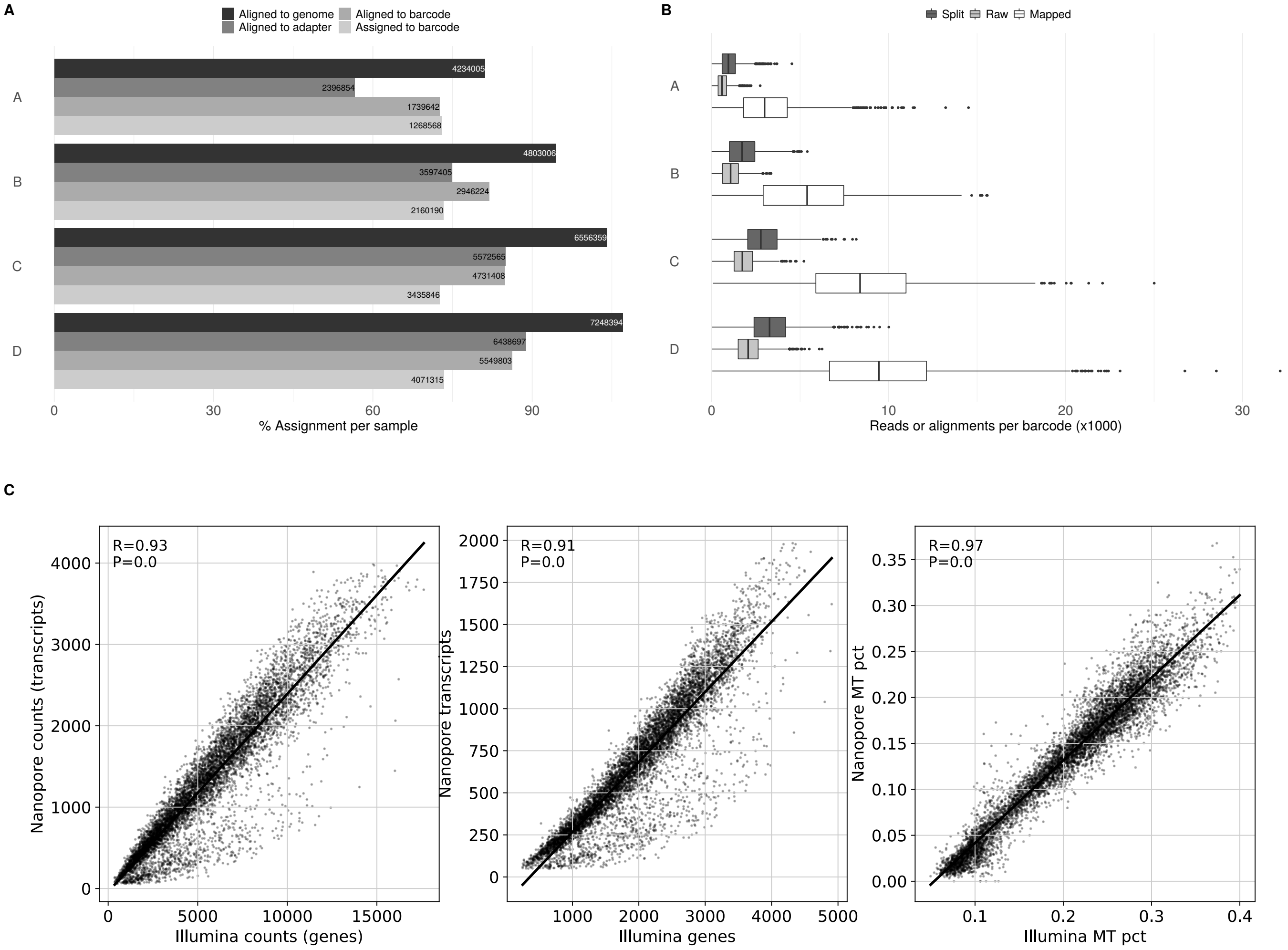

Figure S1. scNaST methodology. A Percentage of assignment by scNapBar at each step of the workflow for each sample (reads shown in bars). Reads aligned to genome are shown as a percentage of total reads. B Number of reads (or alignments) for each sample per spatial barcode. Split alignments are obtained from the genome mapped reads in scNapBar. Raw reads correspond to primary alignments converted to FASTQ format. Mapped are alignments to the transcriptome used for transcript abundance quantification. C Scatter plots showing the correlation between read counts, genes or transcripts, and the percentage of mitochondrial reads between Illumina and Nanopore libraries for all four samples, using the intersection of common spatial barcodes after quality filtering.

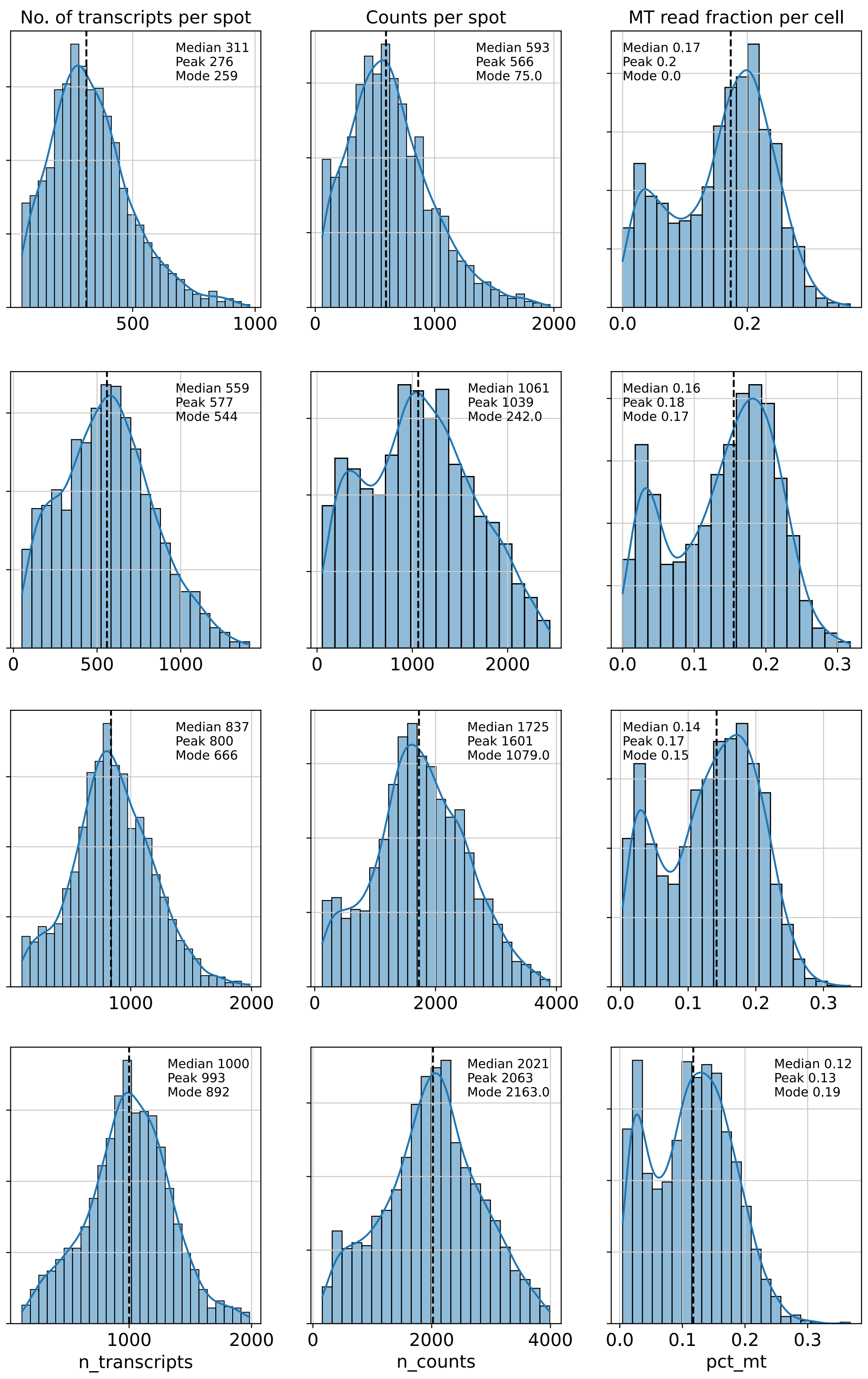

Figure S2. Quality control (Nanopore). Distribution of number of transcripts, counts, and mitochondrial fraction per spatial spot after assignment and quality filtering, for each sample. From top to bottom: A, B, C, and D.

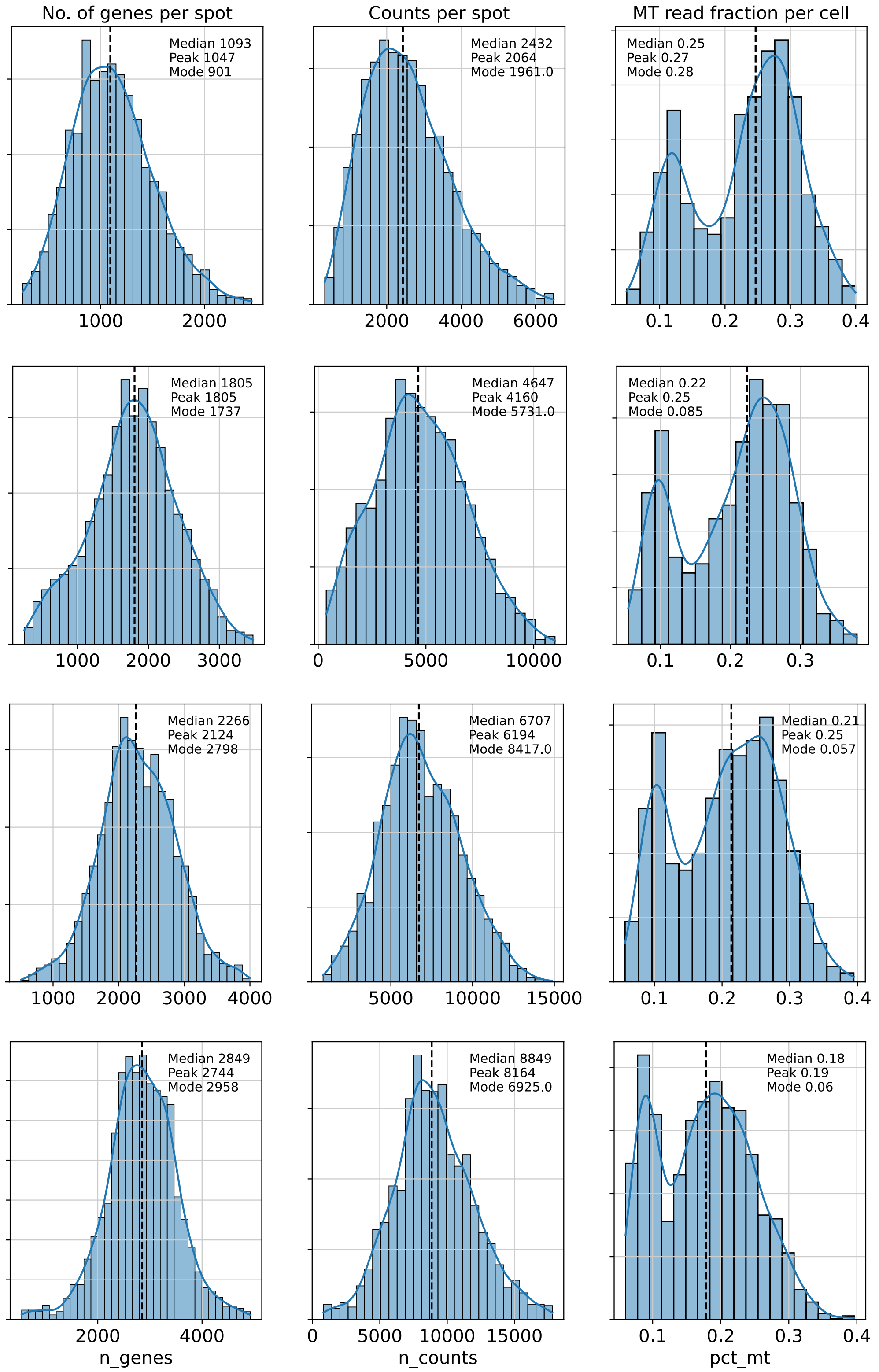

Figure S3. Quality control (Illumina). Distribution of number of genes, UMIs, and mitochondrial fraction per spatial spot after quality filtering, for each sample. From top to bottom: A, B, C, and D.

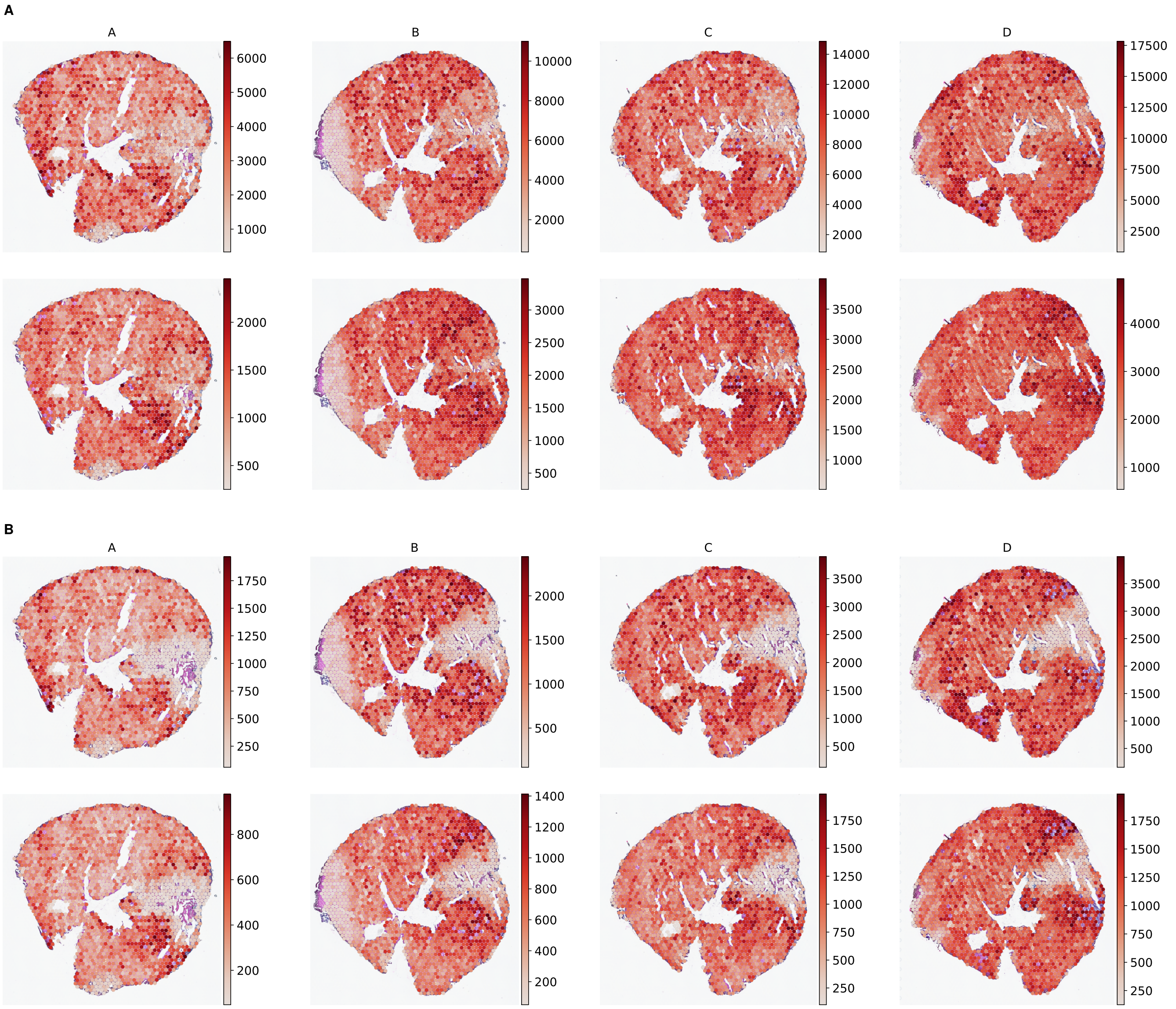

Figure S4. Spatial distribution of UMIs or counts (top) and genes or transcripts (bottom), for each A Illumina and B Nanopore libraries, for each heart slice (from left to right).

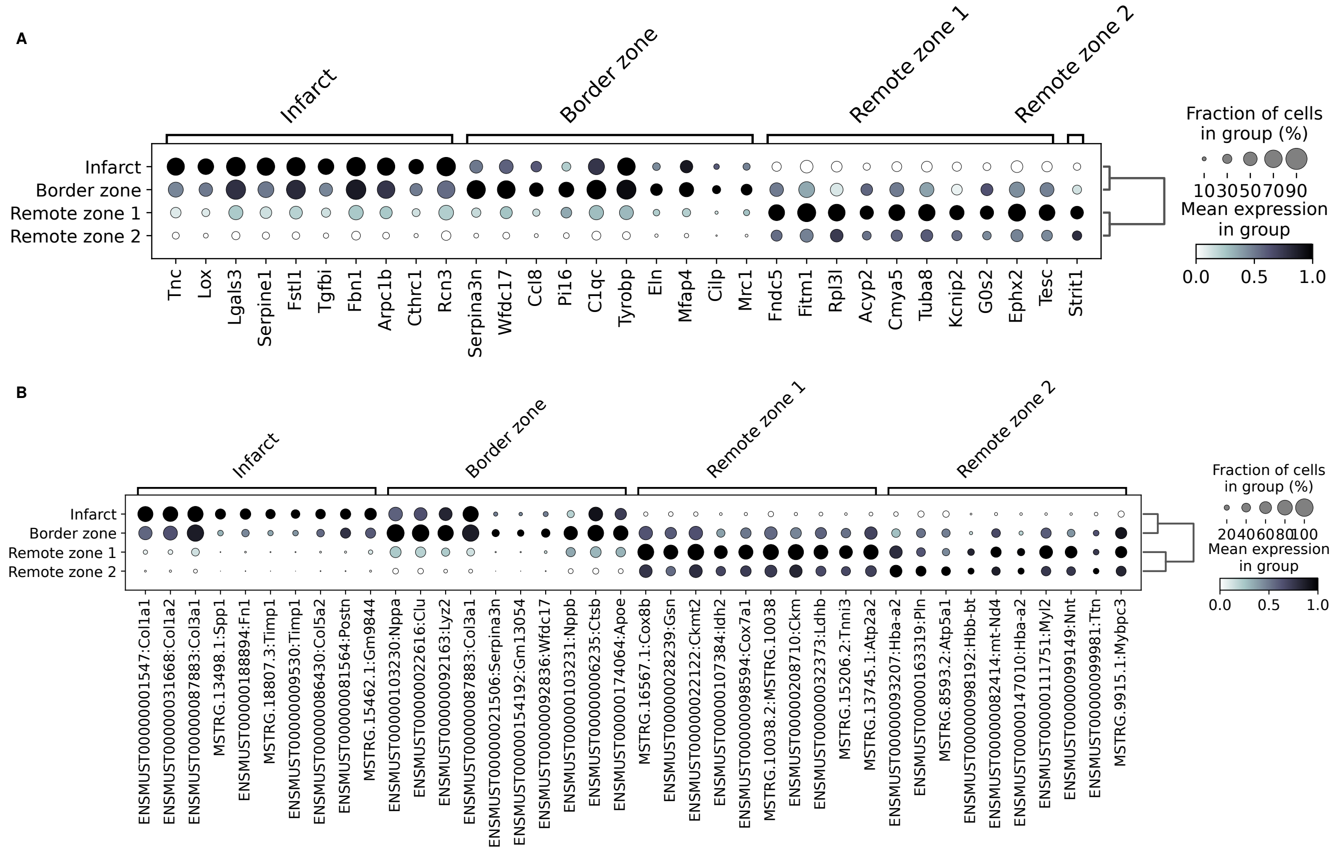

Figure S5. Dot plot of top markers of each regions for each A Illumina and B Nanopore libraries. The top markers were filtered based on log fold change (0.2) and fraction of genes expressing the gene within the region (0.2).

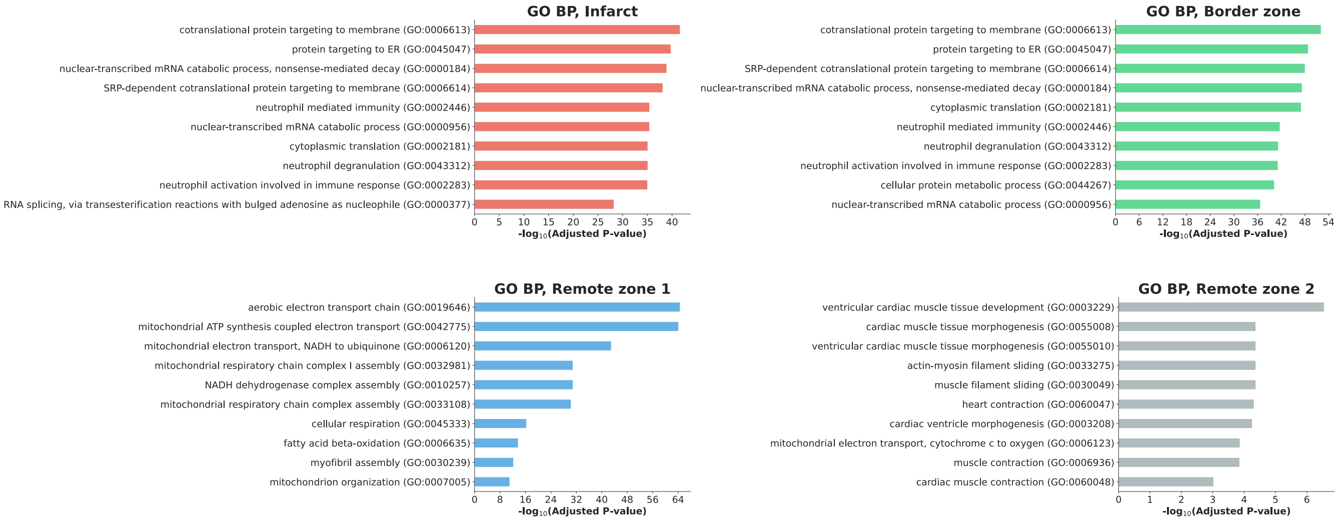

Figure S6. Overrepresentation analysis of biological processes among markers that are spatially variable in each region. Markers were identified using a using a Wilcoxon rank sum test with Benjamini-Hochberg correction. Spatially variable genes were identifed for each sample using SPARK.

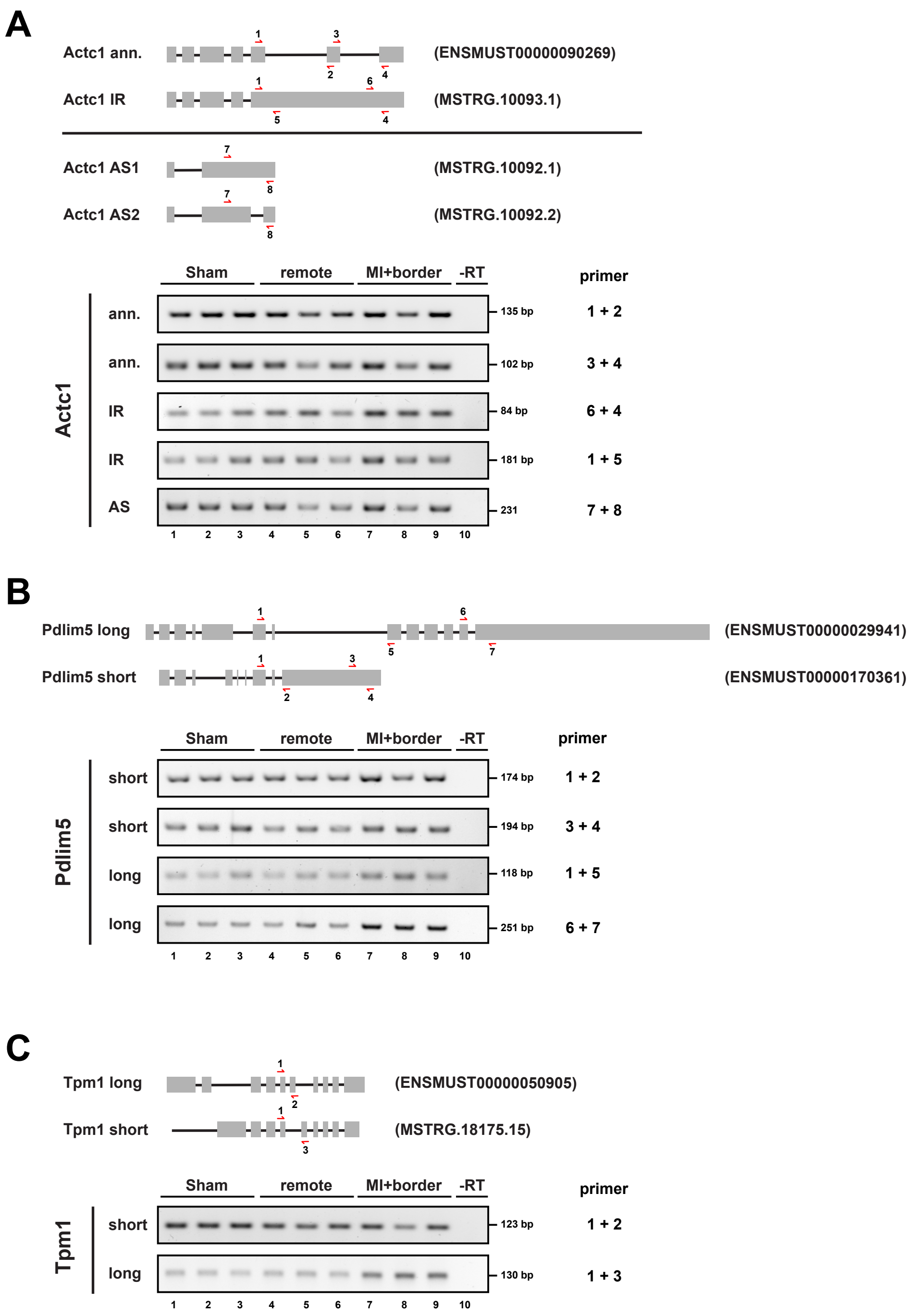

Figure S7. Validation of selected isoform expression in the mouse heart. Total RNA was isolated from the heart of Sham mice or three days post-MI. In the latter case, the RNA from the infarct (including the border zone) and the remote zone was isolated separately. A Upper panel: Schematic representation of the Actc1 isoforms analyzed and primers used for RT-PCR analysis. The expression of annotated (ann.) and intron retention (IR) isoforms, as well as two antisense (AS) transcripts was analyzed. Lower panel: Detection of the Actc1 isoforms with primer pairs as indicated on the right. B Upper panel: Schematic representation of the two Pdlim5 isoforms (long and short) analyzed and primers used for RT-PCR analysis. Lower panel: Detection of the short and long Pdlim5 isoforms with two independent primer pairs as indicated on the right. C Upper panel: Schematic representation of the two Tpm1 isoforms analyzed and primers used for RT-PCR analysis. Lower panel: Detection of the short and long Tpm1 isoforms with primer pairs as indicated on the right.
